# Bacterial cytochrome P450s: a bioinformatics odyssey of substrate discovery

**DOI:** 10.3389/fmicb.2024.1343029

**Published:** 2024-02-07

**Authors:** Gustavo Schottlender, Juan Manuel Prieto, Camila Clemente, Claudio David Schuster, Victoria Dumas, Darío Fernández Do Porto, Marcelo Adrian Martí

**Affiliations:** ^1^Facultad de Ciencias Exactas y Naturales, Instituto de Cálculo, Universidad de Buenos Aires, CONICET, Universidad de Buenos Aires, Buenos Aires, Argentina; ^2^Instituto de Química Biológica de la Facultad de Ciencias Exactas y Naturales (IQUIBICEN) CONICET, Buenos Aires, Argentina; ^3^Departamento de Química Biológica, Facultad de Ciencias Exactas y Naturales, Universidad de Buenos Aires (FCEyN-UBA), Buenos Aires, Argentina

**Keywords:** substrate discovery, functional characterization, genomic analysis, structural features, molecular docking, data-driven analysis

## Abstract

Bacterial P450 cytochromes (BacCYPs) are versatile heme-containing proteins responsible for oxidation reactions on a wide range of substrates, contributing to the production of valuable natural products with limitless biotechnological potential. While the sequencing of microbial genomes has provided a wealth of BacCYP sequences, functional characterization lags behind, hindering our understanding of their roles. This study employs a comprehensive approach to predict BacCYP substrate specificity, bridging the gap between sequence and function. We employed an integrated approach combining sequence and functional data analysis, genomic context exploration, 3D structural modeling with molecular docking, and phylogenetic clustering. The research begins with an in-depth analysis of BacCYP sequence diversity and structural characteristics, revealing conserved motifs and recurrent residues in the active site. Phylogenetic analysis identifies distinct groups within the BacCYP family based on sequence similarity. However, our study reveals that sequence alone does not consistently predict substrate specificity, necessitating additional perspectives. The study delves into the genetic context of BacCYPs, utilizing neighboring gene information to infer potential substrates, a method proven very effective in many cases. Molecular docking is employed to assess BacCYP-substrate interactions, confirming potential substrates and providing insights into selectivity. Finally, a comprehensive strategy is proposed for predicting BacCYP substrates, involving all the evaluated approaches. The effectiveness of this strategy is demonstrated with two case studies, highlighting its potential for substrate discovery.

## Introduction

Bacterial P450 cytochromes (BacCYPs) are heme-containing proteins that, similar to their eukaryotic counterparts, perform oxidation reactions on complex chemical substrates, requiring also molecular oxygen and 2 electrons. In contrast to those from eukaryotes, BacCYPs are soluble proteins and participate in many biochemical pathways. They exhibit an enormous functional diversity and broad capacity to carry out several different oxidation reactions on their substrates, significantly contributing to the generation of a wide range of natural products, many of which have enormous biotechnological potential (Greule et al., 2018). Determining the role of BacCYPs in the production of these metabolites is thus essential to understand their biosynthesis and biotechnological potential. The investigation into BacCYPs is not only a quest to understand their biochemical roles but also a pursuit of their broader implications for various fields. The versatility of these enzymes, with their ability to manipulate diverse substrates, has caught the attention of biotechnologists and pharmaceutical researchers alike. The natural products generated by BacCYPs have not only ecological significance but also hold the promise of yielding new therapeutic agents, industrial chemicals, and biofuels. Hence, elucidating the intricacies of BacCYP function becomes vital for harnessing their biotechnological potential (Kelly and Kelly, 2013).

The recent exponential growth in the sequencing of different microorganisms has resulted in the annotation of more than 100,000 sequences to this group of proteins, an annotation that, except in exceptional cases, does not go beyond an assignment to the corresponding protein family. In this context, predicting, or at least constraining, *in-silico* the range of potential substrates and reactions catalyzed by each of these proteins, is an interesting but challenging task. Amid the extensive sequencing efforts, the functional characterization of BacCYPs has lagged behind, leaving a considerable gap in our understanding (Khmelevtsova et al., 2017). This is particularly pertinent when it comes to the prediction of substrate specificity and catalytic properties at the molecular level. The emergence of high-throughput structural biology techniques and computational methods offers new opportunities to bridge this gap (Sirim et al., 2010; Bustamante et al., 2016; Darabi et al., 2017). Previous studies from our group and others emphasize the effectiveness of these techniques in target identification (Ramos et al., 2018; Sosa et al., 2018; Palumbo et al., 2022) and predicting interactions between proteins and ligands (Serral et al., 2021). This includes the development of new tools tailored for these approaches, encompassing both sequence (and functional) similarity and physical interactions at the structural level to provide accurate predictions (Arcon et al., 2017; Schottlender et al., 2022).

This study takes advantage of such advances to establish a connection between the sequence-structure variations within BacCYPs and their ability to catalyze specific reactions on a variety of substrates.

Determining specific functional properties or aspects, such as specificity/affinity for certain substrate or catalytic efficiency of a protein with precision and detail at the amino acid level, is not an easy task. However, if protein function is determined by its structure and this, by its amino acid sequence, it should be possible, in principle, to predict protein function from their known structures and sequences (Punta and Ofran, 2008). Therefore, predicting the desired (sequence) specific property (in this case the nature of the substrate for a particular BacCYP), requires knowledge of how this property is related to the known sequence-structure variability.

In the present work, we therefore analyzed the relation between BacCYP sequence-structure variation and its substrate’s chemical nature. With this knowledge we developed a strategy that, starting from the sequence of a given BacCYP, allows predicting the potential substrate of the corresponding BacCYP.

## Computational methods

### Retrieval of cytochrome p450 sequences

To identify all Cytochrome P450 sequences, a search was conducted on the UniProt database using the query protein_name: “Cytochrome P450” and filtered by taxonomy for “Bacteria (Eubacteria) [2].” This comprises the large whole BacCYP set. Secondly, we filtered for “proteins with 3D structure” in order to obtain proteins with available structure in the PDB. This set corresponds to the BacCYP structure data set. Finally, we also filtered for “proteins with catalytic activity” to find those BacCYP450s with annotated chemical reactions and their respective substrates and products. Furthermore, we enhanced this search by gathering additional information from the KEGG database (Kanehisa and Goto, 2000). This corresponds to the Known Reaction (KR) CYP dataset. Table 1 shows the number of sequences in each dataset. To perform the subsequent analysis, only the sequence segment corresponding to the CYP450 domains were retained. Domains were defined according to CYP450 PFAM HMM and manipulated using HMMER.

**Table 1:** Numbers of bacterial CYPs sequences.

All BacCYPs sequences	BacCYPs with experimental structure	BacCYPs with known reaction
118,225	133	120

### Multiple sequence alignment (MSA) and phylogenetic tree reconstruction

Multiple sequence alignments (MSAs) were generated using MAFFT (Katoh et al., 2002), with incorporation of structural data from the Protein Data Bank (PDB) (Berman et al., 2000; Katoh et al., 2002) by applying the –dash command. Informative blocks for phylogenetic analysis were selected with BMGE (Criscuolo and Gribaldo, 2010) and the BLOSUM62 matrix. To perform phylogenetic analysis, we selected a representative sample of 2,000 sequences from the entire universe of known BacCYPs using Cd-hit (Li and Godzik, 2006), with an identity threshold of 0.4. Phylogenetic trees were constructed using PhyML (Guindon et al., 2010). The optimal models were obtained using the Smart Model Selection (SMS) approach (Lefort et al., 2017). The groups of proteins were obtained using hclust (Hierarchical Clustering) based on a distance matrix computed with the cophenetic function of the R package ape (Paradis and Schliep, 2019). The number of clusters were defined in order to maximize the number of groups that contain at least 1% of the total sequences.

#### Searching for substrates and/or ligands that bind to BacCYPs

We retrieved substrates involved in the catalytic activity of BacCYPs from the Rhea database (Bansal et al., 2022). Ligands that bind to BacCYPs in crystallized structures were obtained from the Protein Data Bank (PDB), and filtered for biologically relevant ligands using MOAD, while those compounds that showed activity in binding assays were extracted from the ChEMBL database (version 32) (Mendez et al., 2019). We only kept ligands with a pchembl value (a measure of the ligand affinity) (Bento et al., 2013) greater than 6. Finally, additional BacCYP substrates and products were retrieved from annotated reactions within the KEGG database. For performing chemical structure processing, compound structures were stored in SMILES format.

#### Grouping substrates by similarity

Groups of substrates were made using the Butina Clustering method (Butina, 1999), which is implemented in the open-source cheminformatics software RDKit. We evaluated different thresholds to obtain a balance between an appropriate number of clusters and a small number of unclustered ligands.

#### Creation and evaluation of structural models built with Modeller and AlphaFold

To create protein models using Modeller (Sali and Blundell, 1993), we selected a sample of 12 proteins with crystallized structures, to test the model accuracy using templates displaying different phylogenetic distances. For each protein, we selected three templates with short phylogenetic distances (0–2), three with mid-distance (2–4), and three with long distances (>4). We created ten replicas for each model and selected the best one based on its DOPE score (DOPE, Discrete Optimized Protein Energy, is a statistical potential that estimates the energy of a given protein model and assesses its quality) (Shen and Sali, 2006).

We evaluated each model against the reference experimental structure by computing their whole domain RMSD, Alpha Carbon RMSD (CA-RMSD), and active site RMSD.

For the AlphaFold (Jumper et al., 2021) models, we used local colabfold v1.3.0 (Mirdita et al., 2022), an older version that uses the pipeline of AlphaFold v2.0, which has the model weights optimized using training data from the PDB released until April 30, 2018. To avoid possible biases from crystal structures used to train the model, we selected 18 BacCYPs whose crystal structures were released after the mentioned date. For each protein, we generated 5 models without templates. We selected the best model based on the pLDDT score (the default metric used by AlphaFold to rank models). Finally, we evaluated them in the same way as the previous homology based models by comparison with the reference structure.

#### Molecular docking

To perform docking assays, we selected a set of ligands that included, at least, two compounds from each substrate group (the ligand set). For each ligand group, we selected a representative case of a PDB crystallized protein known to bind to the group, and subsequently performed docking simulations with the entire set of ligands. Grids were built using AutoGrid, with a grid size of 40 × 40 × 40 Å for ligands with fewer than 35 atoms and a grid size of 60 × 60 × 60 Å for ligands with more than 35 atoms. The grid spacing was set to 0.375 Å, and the grid center was positioned 10 Å above the heme group. Docking calculations were performed with AutoDockGPU (Santos-Martins et al., 2021) using the following parameters: ga_run = 100, rmstol = 2.5 Å.

#### Biased docking

In the present work we used the Bias docking protocol, a custom script-based method developed previously by our research group (Arcon et al., 2019). Biases are applied as energy rewards to specific ligand atoms involved in relevant and previously known protein-ligand interactions. To identify the most appropriate sites for introducing the corresponding biases, we analyzed critical BacCYP-ligand interactions in known complexes. We introduced a bias for ligands coordinating the heme group, and up to 3 additional biases based on the observed interactions.

#### Genetic context search

We developed a custom python script that queries the KEGG database for nearby genes in the genome of the target organism up to a specified genomic distance threshold. The script retrieves those proteins located up/downstream the target gene, and extracts annotations on reactions involving products and substrates (from the MODULE section), as well as their corresponding Enzyme Commission (EC) numbers. We also searched for the corresponding up/downstream proteins information of their catalytic activity and/or ligand binding activity in KEGG, PDB and ChEMBL. We computed the Tanimoto Index of similarity between the known substrates and products from BacCYPs and from nearby genes using RDkit from their corresponding SMILES format.

## Results

The results are presented as follows: first, an updated analysis of the diversity of BacCYPs sequences and structures is presented, along with an evaluation of the quality and applicability of homology-based and alpha-fold models. Subsequently, the substrate diversity is analyzed in a phylogenetic context, while considering the use of genetic context to infer potential BacCYP substrates. Thirdly, the molecular docking capability is examined to determine the BacCYP-substrate complexes and to differentiate the possible substrate from other ligands. Finally, a BacCYP substrate prediction scheme is devised and evaluated, with a consideration of its potential applicability based on the available information.

### Sequence and structural diversity of BacCYPs

**Figure 1:**
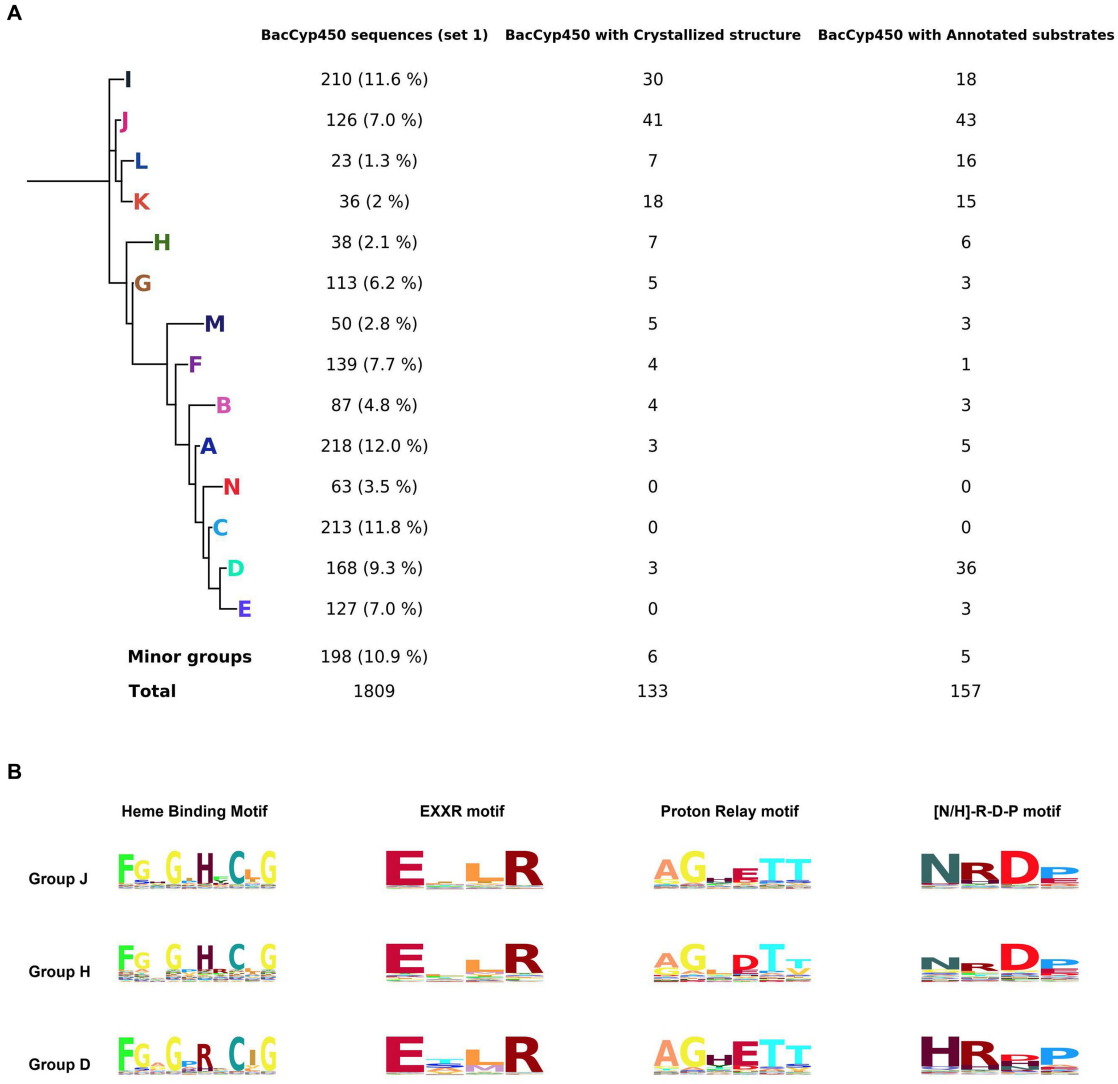
Phylogenetic tree representing the major groups of BacCYPs of a sample that represents the full universe of this protein class (set 1), indicating the number of proteins in each group that have annotated structures and bound substrates or ligands **(A)**. Configuration of groups J, H, and D, with their corresponding Hidden Markov Model (HMM) logos highlighted, reveals distinctions in the H/R amino acid from the sixth position of the Heme Binding Motif, variations in the D/E amino acid at the fourth position of the Proton Relay Motif, and differences in the N/H amino acid at the first position of the [N-H]-R-D-P motif **(B)**.

We begin our analysis looking at the current BacCYPs sequence diversity, performing a MSA of 1809 sequences defined as set 1 (see methods), which includes structural information from 202 representative crystal structures. After removing low information blocks, we obtained a complete phylogenetic tree (Figure 1A) and the associated HMM. Based on this tree we classify the BacCYPs in 14 different monophyletic groups, 11 of them with at least one representative structure.

The MSA and HMMs evidence those sequence motifs conserved in all BacCYPs (Representative examples shown in Figure 1B, and detailed results displayed in Supplementary Figure S1) as well as the sites that are specific for each group (Table 2).

**Table 2:** Group-specific conserved amino acids and their respective positions in the BacCYP domain.

BacCYP group	Distinctive conserved features
A	–
B	L65, W171, E360
C	P121, T168
D	–
E	–
F	–
G	A128
H	W155
I	G116, A128, D149, W155
J	D121, A128, P144, W155
K	P131, L132, P133, I137, P144
L	P131, L132, P133, I137, P144, R149, W155, S156
M	P162
N	–

The length of the BacCyp domain exhibits enormous variation, with an average core length of 403 residues. Within this domain, 33 positions demonstrate high information content. Among conserved features are: (i) the heme binding motif (positions 364-373) F-[G/S]-X-G-X-[H/R]-X-C-X-G in which the last position (G) corresponds to A in group N; (ii) the EXXR motif at positions 292-295; (iii) the proton relay motif, position 236-241 [A/G]-GX-[D/E]-T-[T/S] (Werck-Reichhart and Feyereisen, 2000); and (iv) positions 332-335 presenting the [N/H]-R-D-P motif which differentiates groups at A, B, C, D, E, F, M and N displaying mostly a Histidine, from the others which display mostly an Asparagine.

**Figure 2:**
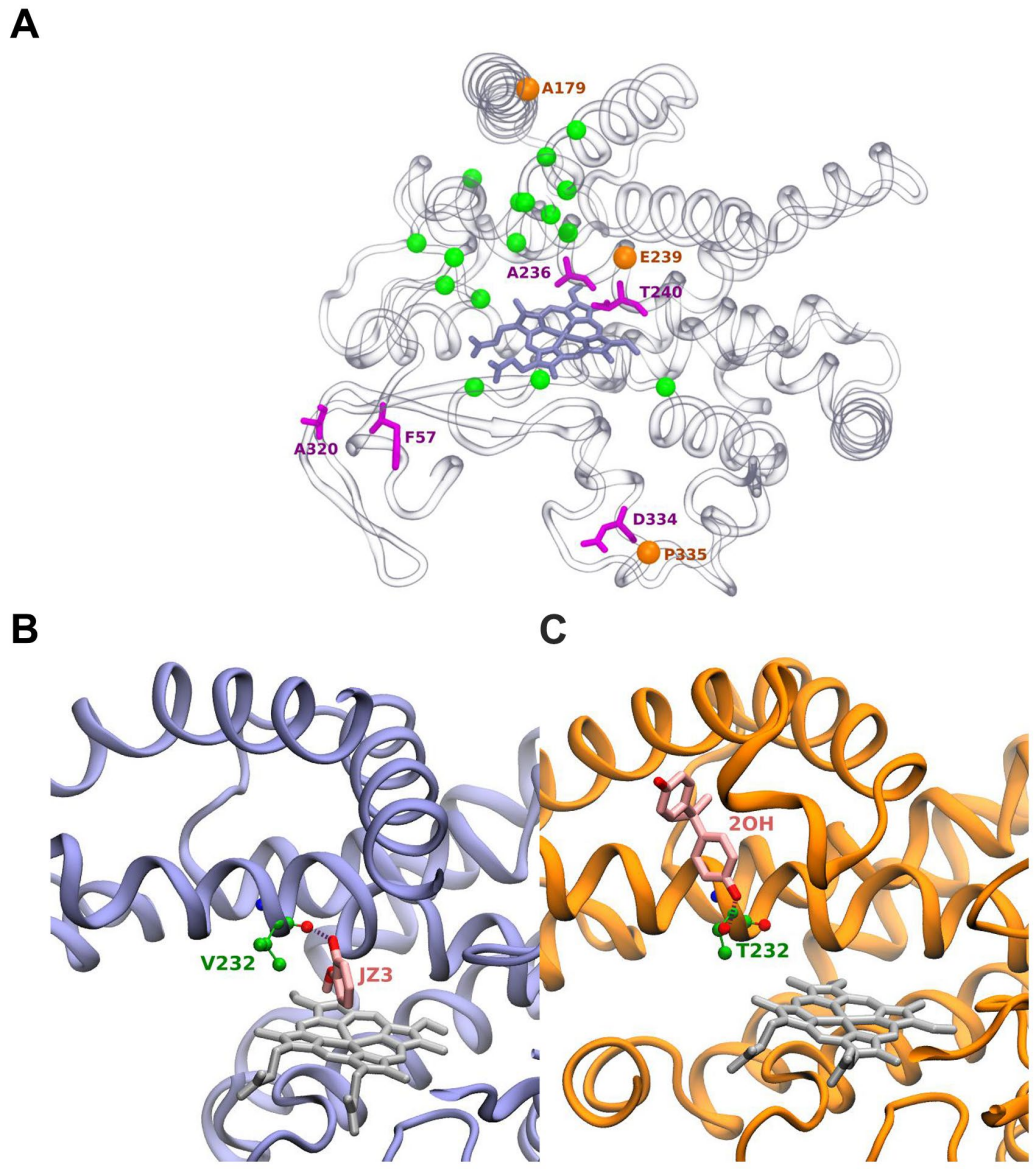
Structure of BacCYPs highlighting active site residues. Residues shown as magenta sticks correspond to most conserved residues. Orange spheres correspond to positions of conserved positions where nonetheless different residues are observed establishing ligand interactions. Green spheres correspond to remaining non-conserved active site residues **(A)**. Structure of GcoA from *Amycolatopsis* sp. (strain ATCC 39116 / 75iv2) bound with Guaiacol (PDBid JZ3) highlighting its interaction with the Val232 backbone **(B)**. Cyp7863 from *Streptomyces peucetius* bound with Bisphenol A (PDBid 2OH) highlighting its interaction with the Thr232 side chain **(C)**.

The active site of BacCYP is composed of approximately 24 residues (Figure 2A). Among these residues, 5 (shows as magenta sticks) display a notable level of conservation, underscoring their pivotal role in the enzymatic function. Particularly, position 57 is predominantly occupied by either phenylalanine (F) or tyrosine (Y). Position 320 is predominantly occupied by alanine (A) or lysine (K). Furthermore, the proton relay motif encompasses positions 236 and 240, which are generally occupied by alanine (A) and threonine (T), respectively. Lastly, position 334 from the [N/H]RDP motif is commonly occupied by aspartic acid (D).

Interestingly, in certain PDB structures, it is observed that amino acids involved in ligand interactions from highly conserved positions (orange spheres) 179 (mainly A), 239 (typically E or D) from the proton relay motif, and 335 (usually P in the [N/H]RDP motif) are substituted by less common amino acids (D, H, and D, respectively). Highlighting the plasticity of particular BacCYPs to bound specific ligands. The remaining 16 residues (green spheres) in the active site exhibit high variability. This observation aligns with the versatile nature of BacCYP enzymes, allowing them to accommodate a diverse range of substrates. As an illustrative example, at position 232, the BacCYP GcoA from *Amycolatopsis* sp. (strain ATCC 39116 / 75iv2) presents a Valine, which interacts through its backbone with the ligand Guaiacol (PDB ID JZ3) (Figure 2B). In contrast, in Cyp7863 from *Streptomyces peucetius*, residue corresponds to a Threonine, and its functional group establishes an hydrogen bond with a distinctly different compound, Bisphenol A (PDB ID 2OH) (Figure 2C). Concerning the group distinctive characteristics most of them are not related to the active site, and thus are not expected to drive a group based substrate specificity. Moreover, analysis of all available BacCYPs structures in complex with substrates reveals that none of the relevant protein-ligand interactions corresponds with groups specific residues and thus protein-ligand are very sequence specific.

We now turn our attention to structural coverage. Mapping of all available BacCYPs structures on the phylogenetic tree, shows that there is at least one structure for most of the major groups. The mean Ca-RMSD between structures of the same group is about 1.51 Å (ranging from 0.262 Å to 2.703 Å), and increases to 2.52 Å (varying from 1.517 Å to 4.579 Å) when structures from different groups are compared. Clearly there is significant structural variation, even inside the same phylogenetic group.

**Figure 3:**
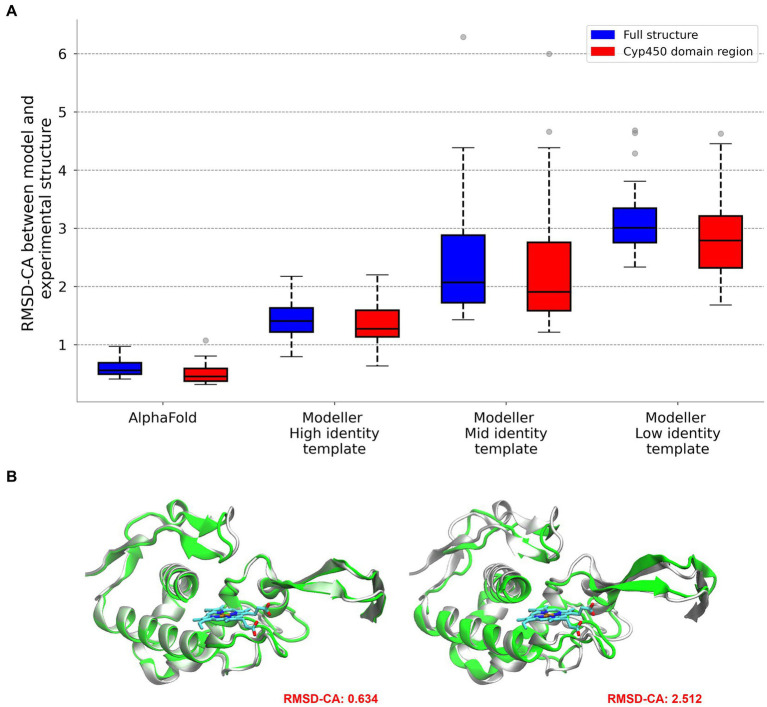
Comparison between reference and modeled structures using AlphaFold and Modeller. RMSD values are provided for the entire protein and for the specific region corresponding to the BacCYP domain **(A)**. Visual representation for the comparison of high and low quality models **(B)**.

To analyze the potential structural coverage using homology based models, we analyzed the model quality and precision as a function of sequence diversity. We selected 12 structures as test cases (see methods) and for each we built 9 models, three using a high identity template (sequences in the same subgroup), three of moderate identity (sequences in the same group) and three with low identity (using templates from a different group). The resulting models were evaluated by comparing with the corresponding real structure (see methods). We also built 20 structures using AlphaFold. We were particularly careful to select those that were deposited in the PDB after the cut-off date used for training the model. The results, presented in Figure 3, show, as expected, that models built with high identity template, more than 90% of structures are modeled with an overall CA-RMSD below 2 Å, and high QMEAN, which is an excellent prediction. Value falls to little more than half the structures for moderate identity templates. Low identity templates show RMSD values which are not good enough to trust the structure for further analysis. Most importantly, AlphaFold models are significantly better, even than those modeled with high identity templates. The results from AlphaFold models (and also those corresponding to High identity templates) show that those structures are accurate enough to perform docking calculations, as will be shown later.

### Substrate diversity of BacCYPs

**Figure 4:**
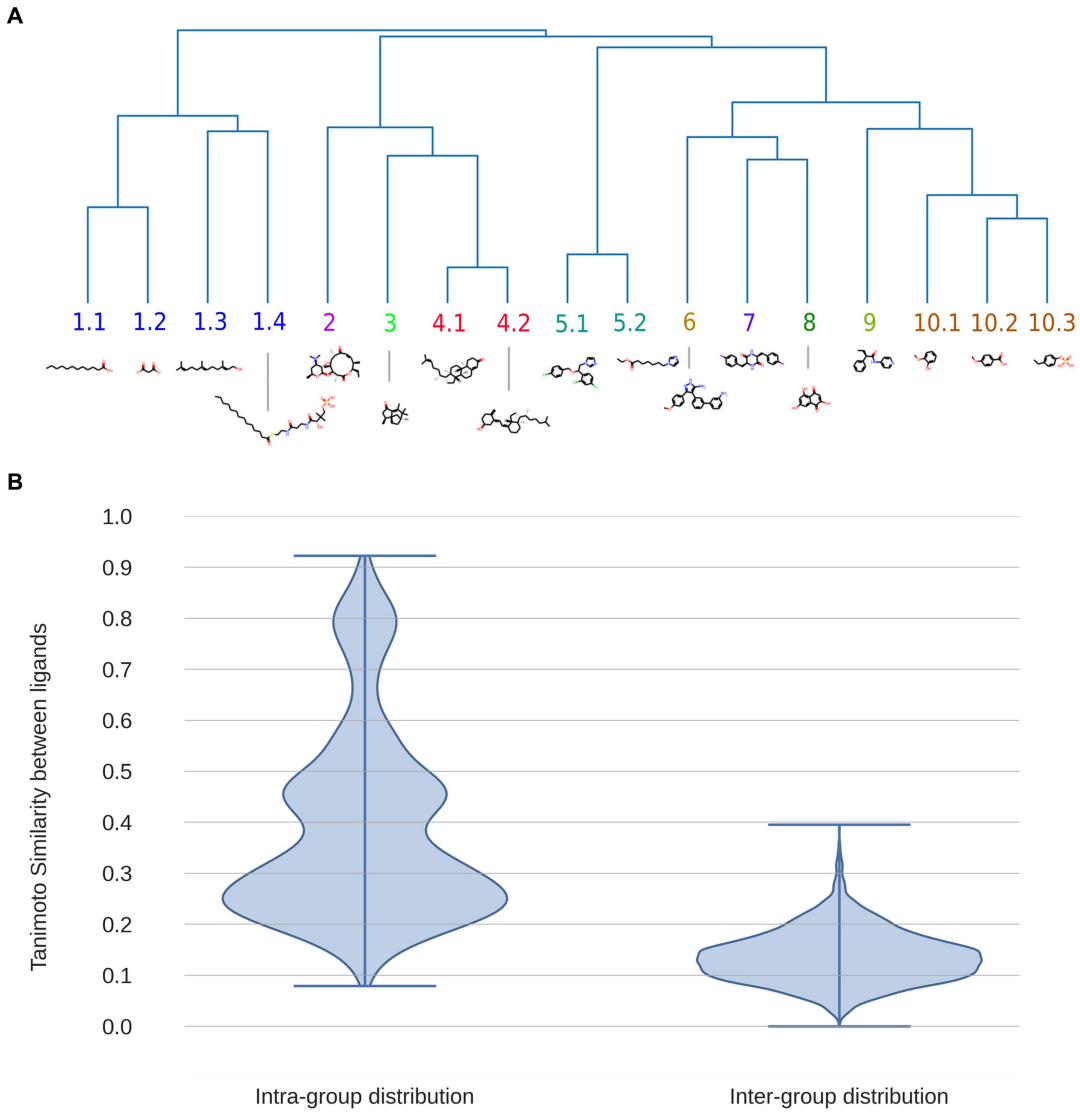
Dendrogram displaying the 17 major groups of compounds bound by BacCYPs, with an illustrative example of each one, where also physicochemical similarity relationships are shown **(A)**. Distributions of Tanimoto Index (similarity) between all possible pairs of compounds within the same group (intra-group distribution) and among compounds from different groups (inter-group distribution) **(B)**.

We begin our analysis of known BacCYP substrate diversity by building a dendrogram of all known BacCYP substrates using Tanimoto Index (TI) to determine their similarity. The results shown in Figure 4A, allow classification of the substrates in 10 major structural groups, showing an overall considerable chemical diversity. Expected groups are those formed by sterol-like compounds (group 4), fatty acid (group 1), camphor and related molecules (group 3) or macrolids (group 2). Group 5, 6, 7 and 9 show more complex structures with several aromatic rings (the complete ligand and group dataset is provided in Supplementary Table S1). To analyze the variance (in terms of TI) of each substrate group we computed the TI between all pairs of substrates within and between groups. The results presented in Figure 4B, shows that TI between groups is very low, while that within groups presents three subclusters. In the first, clearly there are ligand pairs which are very similar (TI > 0.6), in the second ligands with moderate similarity, and then a third large cluster where, even when belonging to the same group, pairs of ligands differ significantly (TI in range 0.15–0.35). Summarizing, the diversity of BacCYP substrates is huge and even if groups of similar ligands can be built, they still present a lot of chemical variation. This huge variation presents major difficulty for predicting a BacCYP substrate with accuracy.

### Relationship between BacCYP phylogeny and substrate

**Figure 5:**
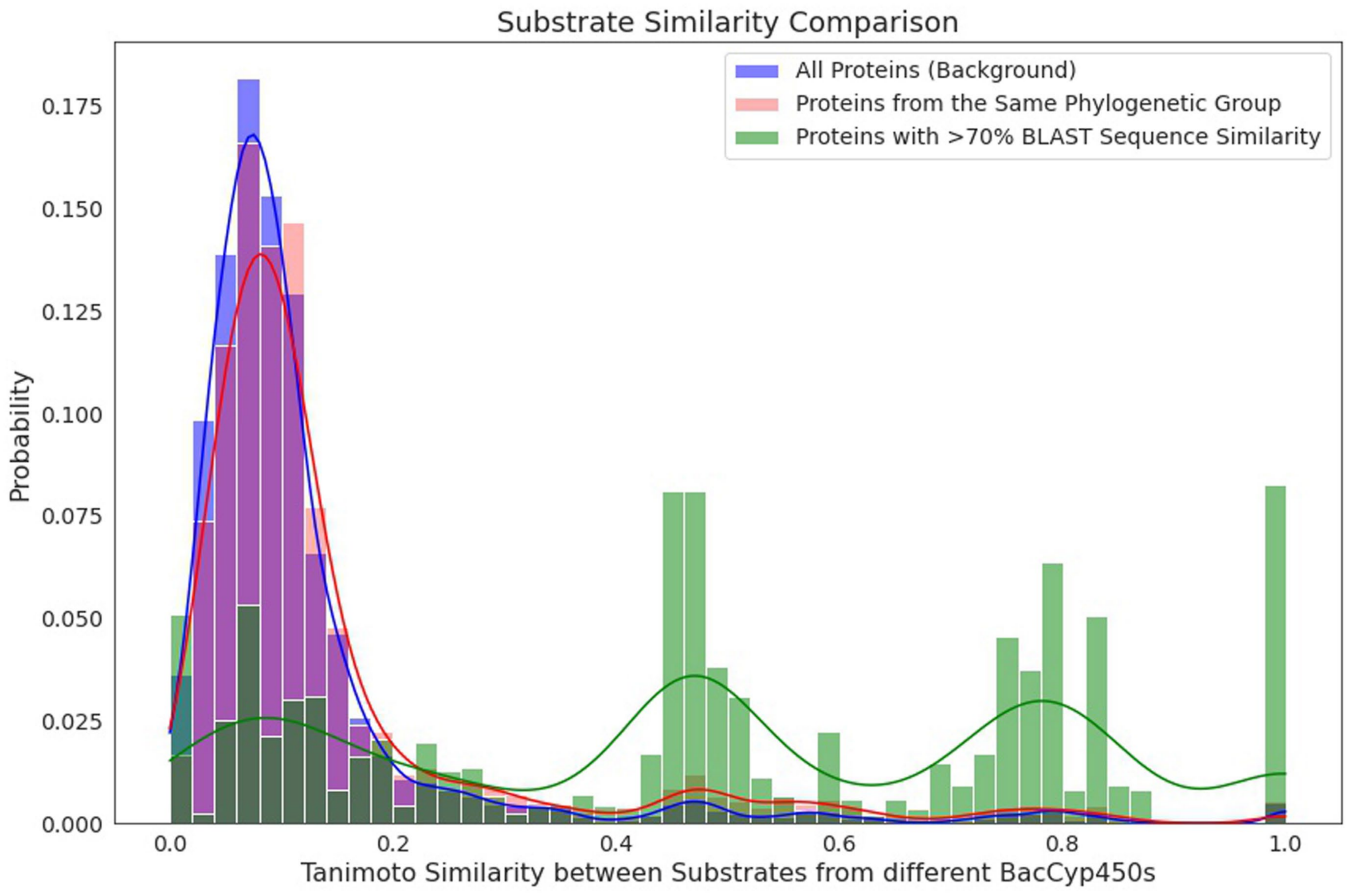
Histograms depicting the similarity by Tanimoto Index between compounds binding to different proteins within the set of all annotated BacCYPs (blue), within the same phylogenetic group (red), and among highly similar proteins, with over 70% identity in BLAST (green).

To analyze the likelihood and reliability of predicting BacCYP substrates (and thus their potential reaction) based solely on sequence comparison, we firstly mapped the known substrates on the phylogenetic tree. As expected, given that phylogenetic groups are very diverse, they do not provide clues about the substrates/products of their corresponding BacCYPs. As seen in Figure 5, the distribution of products/substrates of BacCYPs within the same phylogenetic group (salmon) closely resembles the overall distribution for all annotated BacCYPs (Blue). Interestingly, when examining products and substrates of BacCYPs with over 70% sequence identity (Figure 5 green) the distribution shows peaks at TI values of 0.5, 0.8 and close to 1. These results suggest that although BacCYP substrate cannot be inferred by assigning a particular BacCYP to a given group, similar BacCYPs with known substrate/products may hint to possible substrate of a broader chemical group.

### BacCYP genetic context

In bacteria, metabolic pathways are often organized into operons where the product of one gene is the substrate of an adjacent gene. This arrangement prompted us to investigate whether neighboring genes could be used to infer possible substrates of a given BacCYP. For this sake, we examined available metabolic pathway information for all BacCYPs and their neighbor genes in the KEGG database. For 4,815 BacCYPs we were able to retrieve information about the substrate/product of at least one of their neighbors, but only for 120 of these BacCYPs the reactant and/or product are known. We computed the chemical similarity index between the substrates/products of the adjacent gene and those reported for the BacCYPs. Consistent with our idea, the precise BacCYP substrate/product can be retrieved by adjacent genes in almost 80% of the cases where results are identified. Expanding the search to genes up to 2000 bp apart enables us to obtain information for a greater number of BacCYPs, albeit with a higher number of potential compounds.

**Figure 6:**
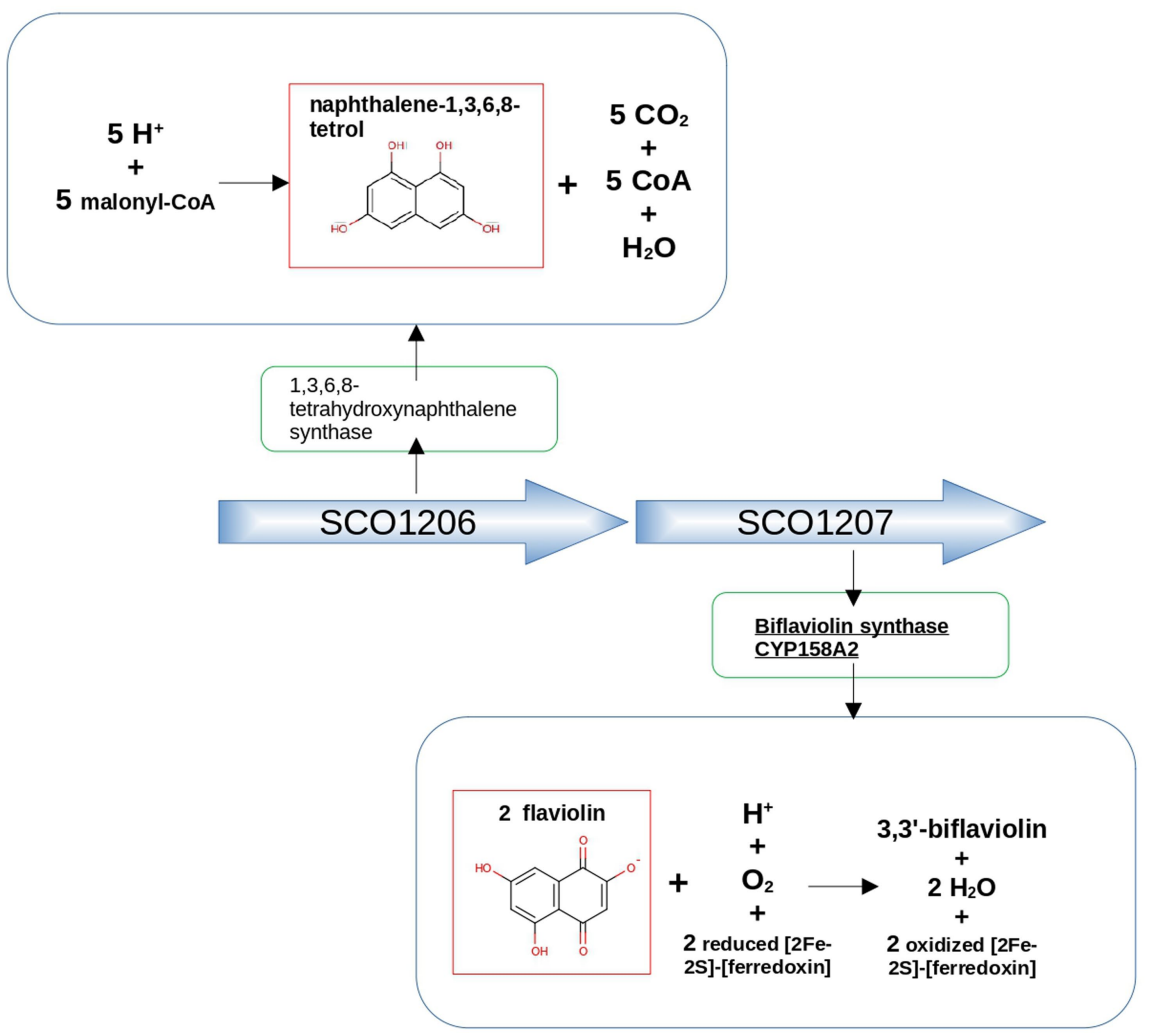
Genetic context approach illustrated using the example of neighboring genes SCO1206 and SCO1207 in *Streptomyces coelicolor*. SCO1206 encodes 1,3,6,8-tetrahydroxynaphthalene synthase, while SCO1207 encodes Biflaviolin synthase CYP158A2. The figure highlights the structural similarity between naphthalene-1,3,6,8-tetrol, a product of the reaction catalyzed by 1,3,6,8-tetrahydroxynaphthalene synthase, and flaviolin, the substrate of the protein encoded by the adjacent gene, Biflaviolin synthase CYP158A2.

As an illustrative example, Cyp158A2 from *Streptomyces coelicolor* (Uniprot ID Q9FCA6) synthesizes 3,3′-biflaviolin using flaviolin as its substrate. Among the two proteins encoded by adjacent genes, one is a 1,3,6,8-tetrahydroxynaphthalene synthase (Uniprot ID Q9FCA7), and the other a Cupin type-1 domain-containing protein with Uniprot ID Q9FCA5. The first one produces the compound naphthalene-1,3,6,8-tetrol, which shows a similar structure to the mentioned substrate of Cyp158A2 (see Figure 6). This finding underscores the typically frequent coordination facilitated by operons in bacteria, where the synthesis of CYP450 enzymes closely aligns with the production of partner proteins resulting in a coordinated expression of a metabolic module.

### Docking prediction of BacCYP-substrate structure

**Figure 7:**
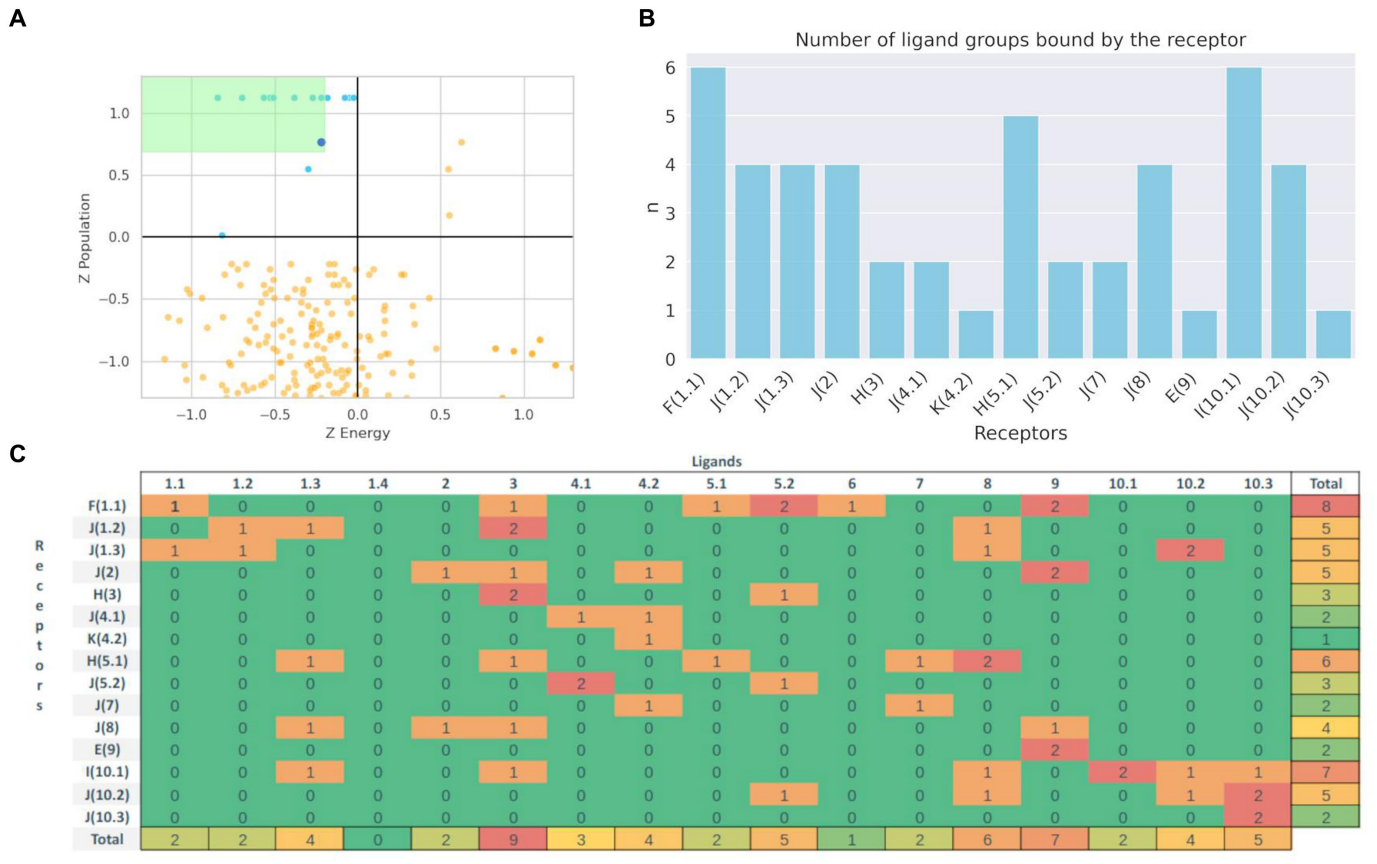
Population vs. Binding Energy plot (Z-score) for the docking of true (Blue) and other (Orange) ligands. Green zone represents the zone that defines potential binders **(A)**. Distribution of the number of predicted 17 different group ligands that bind to each Phylogenetic defined type of BacCYP **(B)**. Binding results matrix. Rows represent Representative Ligand Group-Bound Proteins, with the actual known ligand group that binds the corresponding protein between parenthesis, columns represent each ligand type group. Numbers represent the count of ligands from the respective groups that are predicted to bind the corresponding BacCYP according to the docking results **(C)**.

To analyze whether molecular docking could be used to select potential substrates of a given BacCYP, we analyzed the pose prediction and selectivity performance of Autodock-bias for a representative set of BacCYPs against compounds from all ligand groups. Specifically, we selected 15 protein structures, each of which binds to a ligand from a different group, referred to as Representative Ligand Group-Bound Proteins (RLPs, detailed in Supplementary Table S2), and 35 test ligands (two for each group, except group 5.1 with 3 ligands), which lead to an overall of 525 docking calculations. Each predicted pose was characterized in terms of both its estimated binding energy and population, normalized as Z-scores. As shown in previous works from our group analysis of both energy and population for each docked protein-ligand pair allows to select those poses which are outliers (i.e. those with negative binding energy and high population) in the upper left quadrant of the corresponding 2D plots as positives (i.e. potential binders). The results are presented in Figure 7. It is important to note that, as observed earlier, there is considerable substrate diversity within the same group of BacCYPs. Consequently, due to limited available information, we selected proteins from recurring phylogenetic groups as RLPs (for example, group J is diverse and well represented in known substrates and crystallized structures, and much information is obtained from proteins from that group). Figure 7A shows the 2D Z-score plot for all relevant obtained poses, the score of the true ligands that bind each BacCYP are shown in light blue and as expected are clear outliers in the upper left quadrant. Comparison of the corresponding predicted poses against reference structures shows, as expected, correct pose prediction with overall average heavy atom RSMDs of 2.143. Clearly, if the correct ligand is found, docking will confirm the BacCYP binding capacity. To analyze BacCYP selectivity we defined as binders those ligands that have Z-scores above 0.9 of the true positive z-score, and present in Figure 7B the number of ligand groups that each BacCYP is predicted to bind. Detailed information on ligand group binding to each RLP is presented in Figure 7C.

The docking results show that some RLPs are more promiscuous than others, with 3 of them being presumed to be highly selective (they are predicted to bind all the test ligands that belong to the same group as their authentic binding compound) and another 4 that positive binders are only ligands from one or two different groups. On the contrary, three RLPs are expectedly highly promiscuous, showing favorable docking results with test ligands from up to six different groups. Detailed analysis shows, for example, that promiscuous RLPs from phylogenetic groups F and H both are predicted to bind quite large test ligands, including a 62 atom long chain fatty acid and a three aromatic ring containing compound. For the RLP from group I, which actual binding ligand (group 10.1) is an aromatic ring ligand with polar substituents, the docking assays suggest a preference for ligands from neighboring groups.

From a ligand centered viewpoint, promiscuous ligands are those from groups 3, 8 and 9. The first two are medium to small size ligands with one to two aromatic rings and polar functional groups. Overall, our docking results show that if a potential substrate is available, docking could support whether or not it can bind, while also providing if no other information is available a selectivity filter that could narrow the range of substrate possibilities.

**Figure 8:**
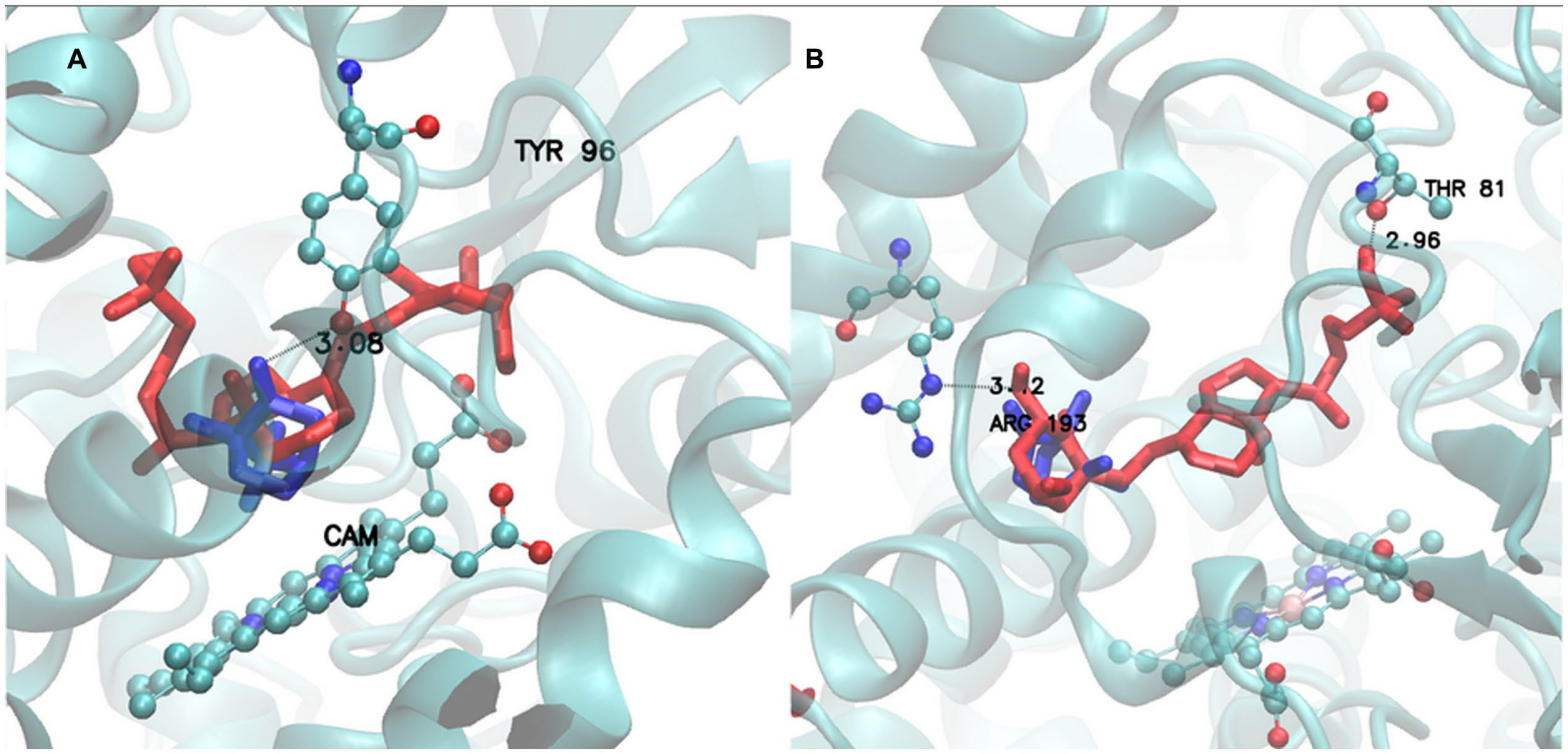
Active site of Group H receptor (1AKD pdb) with CAM (Group 3) ligand (Blue) and VDX ligand (Red) **(A)**. Active site of Group J receptor (5GWE pdb) with CAM (Group 3) ligand (Blue) and VDX ligand (Red) **(B)**.

Finally, a visual example of BacCYP specificity is provided in Figure 8. Left panel shows a BacCYP from group H (PDB ID 1AKD) with its natural ligand (CAM from group 3) in blue. The ligand is rather small and interacts with Tyr96 through a tight hydrogen bond. A larger ligand, VDX (group 10.3) is shown in red, which clearly does not fit the BacCYP active site and indeed clashes with Tyr96. Direct visual comparison is provided in the right panel, where the natural VDX receptor belonging to group J is shown with both ligands using the same color code. To bind VDX this BacCYP (5GWE) displays a larger cavity and is able to establish two hydrogen bond interactions, with Arg193 and Thr81, at each end of the ligand. This BacCYP cannot bind CAM since it lacks Tyr96 and its cavity is too big (docking results in many different poses with low populations). Clearly, and as expected, BacCYP specificity is directly linked to active site size and the presence of specific residues that are able to interact with the ligands.

### Integration of the proposed methodologies into a predictive framework

**Figure 9:**
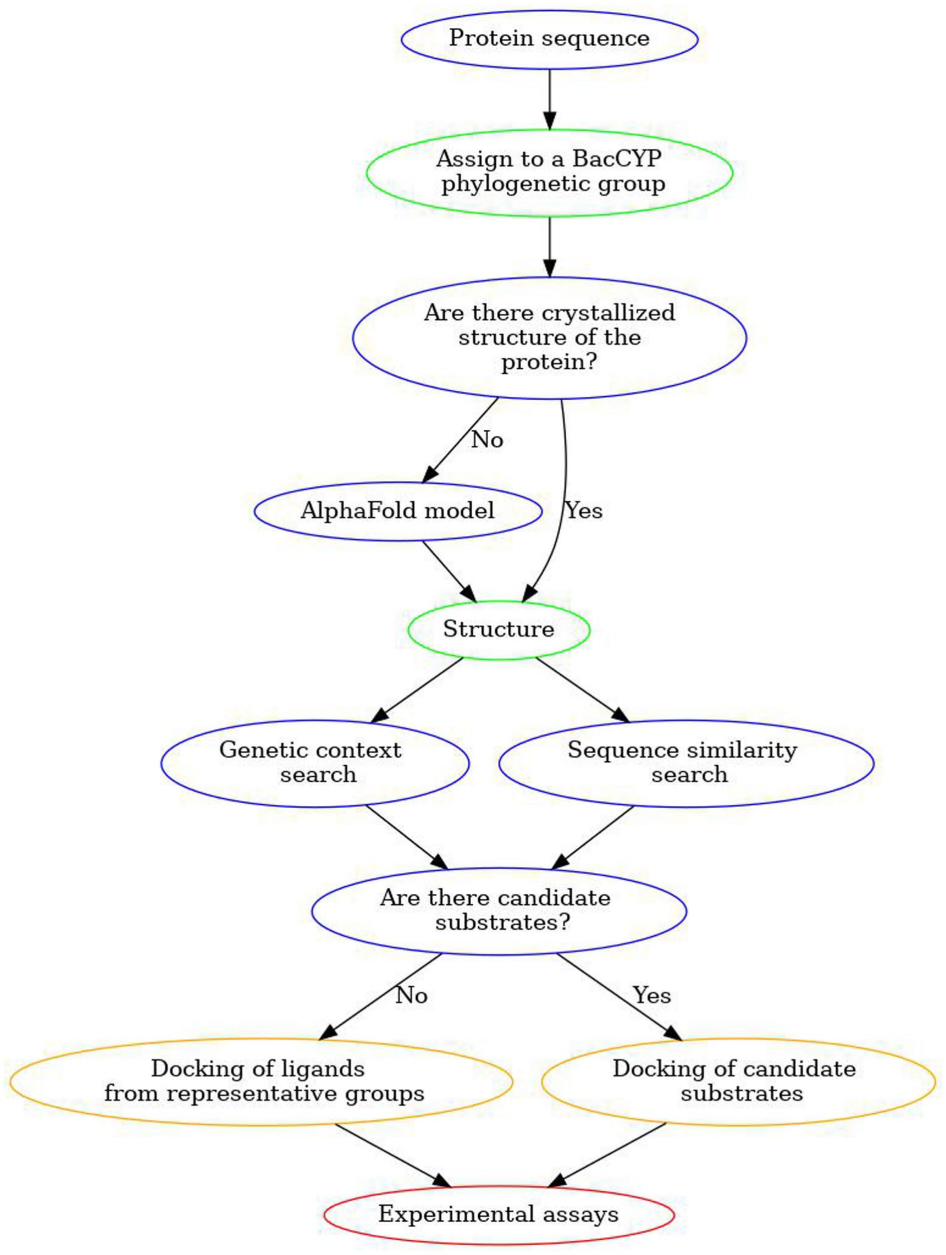
Scheme of our proposed workflow to discover substrates for unannotated BacCYPs.

To show the predictive capacity of the designed strategy, we applied all the described steps to two BacCYPs with known substrates. The overall strategy is shown in Figure 9 and can be briefly described as follows. Starting with the sequence of a new BacCYP, it is assigned to one of the 14 phylogenetic groups, and the group promiscuity is evaluated. A search for structures in the PDB is conducted, and if a relevant structure exists, it is utilized; otherwise, either an AlphaFold model is generated, or, if a very similar protein structure is available, an homology model is built. BacCYP neighboring genes are retrieved and if their substrates/products are annotated they are used as potential binders. Finally docking is performed for representative members of each ligand group and the potential binders. A positive docking of a potential binder is a strong indication that the potential substrate (or a similar chemical entity) is found. Following, we present an application of the described strategy to two example cases.

**Figure 10:**
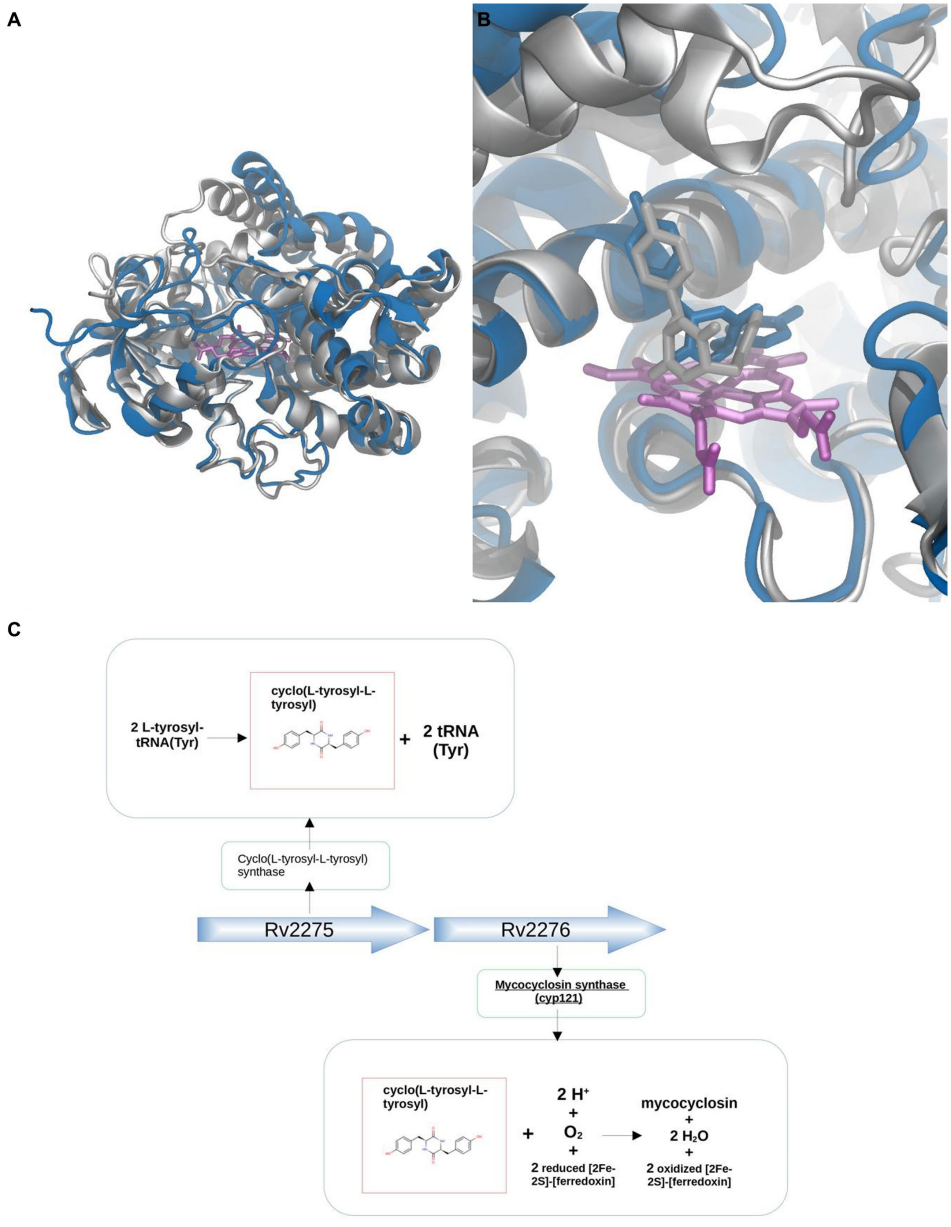
Substrate discovery workflow applied to MtCYP121. Comparative analysis between the X-ray (silver) and Modeller generated (blue) structures **(A)**. Comparison between X-ray (silver) and docked pose (blue) for the natural Cyclo(L-tyrosyl-L-tyrosyl) ligand **(B)**. Genetic context approach showing that Cyclo(L-tyrosyl-L-tyrosyl), substrate of CYP121, is the product of the protein Cyclo(L-tyrosyl-L-tyrosyl) synthase, encoded by the adjacent gene Rv2275 **(C)**.

The first example case corresponds to CYP121 of *Mycobacterium tuberculosis* strain ATCC 25618 / H37Rv (MtCYP121) Uniprot ID P9WPP7, which is involved in the synthesis of Mycocyclosin, and utilizes cyclo(L-tyrosyl-L-tyrosyl) as its substrate (ligand group 7). Sequence analysis shows that MtCYP121 belongs to phylogenetic group J, where ligands from groups 1.1, 1.2, 1.3, 2, 4.1, 4.2, 5.1, 5.2, 6, 7 and 8 are found, clearly phylogenetic analysis does not provide a clear potential substrate. Closest BacCYP with known structure corresponds to mycG from *Micromonospora griseorubida* (Uniprot ID: Q59523) (50% identity) and binds group 2 of ligands. Modeling of the CYP121 structure with Modeller (while we encourage researchers to use AlphaFold, in this particular case of study, we chose Modeller to avoid potential biases stemming from the fact that the actual protein structure was used to train AlphaFold v2.0) using PDB structure 2YGX from protein Q59523 as template results in high quality model (RSMD-CA against known structure of 1.192, shown in Figure 10A).

Analysis of the genetic context shows that genes adjacent to MtCYP121 correspond to Uniprot IDs P9WPF9 (upstream) and P9WLF1 (downstream). The protein P9WPF9 is called Cyclo(L-tyrosyl-L-tyrosyl) synthase, which as the name suggests, synthesizes the mentioned CYP121 substrate from L-tyrosyl-tRNA(Tyr), while the tRNA(Tyr) part from L-tyrosyl-tRNA(Tyr) becomes separated after the reaction (Figure 10C). Docking of the potential substrate Cyclo(L-tyrosyl-L-tyrosyl) results in a clear binder, and thus further supports its positive identification (Figure 10B). It is interesting to note, that docking of the whole set of ligands, results in other two potential types of substrates (from groups 4.1 and 10.2). This result underscores the fact that combining the two aspects, docking and genomic context allows better substrate assignment than any of the two steps separately.

The second case corresponds to Aromatic O-demethylase, cytochrome P450 subunit (Uniprot ID P0DPQ7) of *Amycolatopsis* sp. ATCC 39116 and is quite difficult since no information of neighbor gene substrates/products is available, and its first crystallized structure was released by the PDB in 2018-07-04, after the date used for AlphaFold training. The protein belongs to group I, whose proteins contain substrates from nine different groups, is also quite promiscuous as it is able to bind substrates from groups 10.1 and 10.2, including guaiacol, 3-methoxycatechol, and guaethol. Modeling the structure with AlphaFold version 2.0 yielded a high-fidelity model (RMSD-CA of 0.726 compared to the real structure). Docking results of substrate groups representatives showed that group 10.1 ranked second, just below group 8, while group 10.2 ranked fifth. Although in this case no particular substrate could be defined, structure modeling and docking allowed narrowing the range of substrates to a few groups which include the known binders.

## Discussion

BacCYPs are key proteins of microbial natural product synthetic pathways and therefore, determining their precise role in the production of these metabolites is essential to understand their biosynthesis and biotechnological potential. Moreover, the recent exponential growth in available microbial (or environmental) genomes has resulted in the annotation of more than 100,000 sequences of BacCYPs, an annotation that, except in exceptional cases, does not go beyond an assignment to the corresponding protein family. Clearly, predicting *in-silico* the range of potential substrates and reactions catalyzed by each of these proteins, is an interesting but challenging task.

In the present study we provide three independent approaches for the discovery of novel BacCYP substrates. First, we analyze the BacCYP phylogeny in relation to known homologous protein substrates. Second, we use structure modeling and knowledge based docking to determine which type of ligands fit a given BacCYP active site. And third, we analyze the relation of the known substrates/products of neighbor genes with the BacCYP substrate. We show that all three strategies can contribute to narrowing the range of potential substrates of a given BacCYP. Notingly, phylogenetic analysis is the least informative since similar (by sequence) proteins can bind quite distinct substrates. Docking is quite informative and can be used to discard groups of substrates that clearly do not fit, when no other information is available, or can be used to confirm the binding of a potential identified substrate. In this scenario, we emphasize the significance of our genetic context-based substrate search approach, which has demonstrated a notably high success rate in identifying the correct substrates for BacCYPs associated with neighboring gene reactions. It is important to mention, that as more genes are annotated this information source is only expected to grow thus increasing our method predictive capacity.

To our knowledge there aren’t any works that specifically address BacCYP substrate prediction. However, some previous works analyzed similar strategies as those presented here. For example, the definition of gene clusters encoding proteins responsible for the biosynthesis of various metabolites across a wide range of organisms, including both prokaryotes and eukaryotes is a well known strategy to annotate gene function, including substrate/product specificity (Chavali and Rhee, 2018). Some studies also analyzed gene clusters related to metabolism of broader types of compounds, and among the genes several BacCYPs were annotated (Greule et al., 2018).

Concerning specific BacCYP-ligand interactions, docking and virtual screening strategies have been applied for the study and search of CYP inhibitors, including BacCYPs found in pathogenic bacteria (Reddy et al., 2013; Snow Setzer et al., 2016). Moreover, several works employed structural bioinformatic tools to assess interactions between mutant BacCYPs and compounds of interest (Park et al., 2010).

A separate point concerns the use of AlphaFold models. Our results show that AlphaFold is able to generate accurate BacCYP model structures, even better than those obtained through homology modeling at moderate sequence identity. These accurate models are essential to perform the docking step and thus to our method. The possibility of building models for any new sequenced BacCYP therefore significantly expands their potential annotation using structure driven strategies, which as shown by our results when combined with other coincident information can increase the method’s predictive capacity.

Finally, and from a general point of view, the presented strategy underscores the capacity of bioinformatic methods that leverage on two independent sources of information and their underlying knowledge. First, the vast amount of sequence information and annotated biological databases; and second the use of structure based methods. The level of detail, attained by looking at a protein’s particular tridimensional structure, combined with sequenced guided database annotation retrieval, allows to further annotate the protein beyond family assignment.

## Data availability statement

Publicly available datasets were analyzed in this study. This data can be found at: Uniprot Bacterial Cytochrome P450 sequences: https://rest.uniprot.org/uniprotkb/stream?download=true&format=fasta&query=%28%28protein_name%3A%22cytochrome+p450%22%29+AND+%28taxonomy_id%3A2%29%29. BacCYP binding ligands and PDB structures datasets are included in Supplementary material. Ligands from genetic context search were retrieved from databases mentioned in the manuscript using scripts developed by our group.

## Author contributions

GS: Conceptualization, Data curation, Formal analysis, Investigation, Methodology, Software, Validation, Visualization, Writing – original draft, Project administration. JP: Conceptualization, Data curation, Formal analysis, Investigation, Methodology, Project administration, Software, Validation, Visualization, Writing – original draft. CC: Formal analysis, Methodology, Project administration, Writing – original draft. CS: Formal analysis, Methodology, Project administration, Writing – original draft. VD: Conceptualization, Investigation, Methodology, Project administration, Software, Writing – original draft. DF: Funding acquisition, Methodology, Project administration, Supervision, Writing – original draft. MM: Funding acquisition, Methodology, Project administration, Supervision, Writing – original draft.
