# Supplementary figures and images for: Bacterial cytochrome P450s: a bioinformatics odyssey of substrate discovery
Source: Front Microbiol. 2024 Feb 7;15:1343029. doi: 10.3389/fmicb.2024.1343029 (PMC10879549; doi:10.3389/fmicb.2024.1343029)

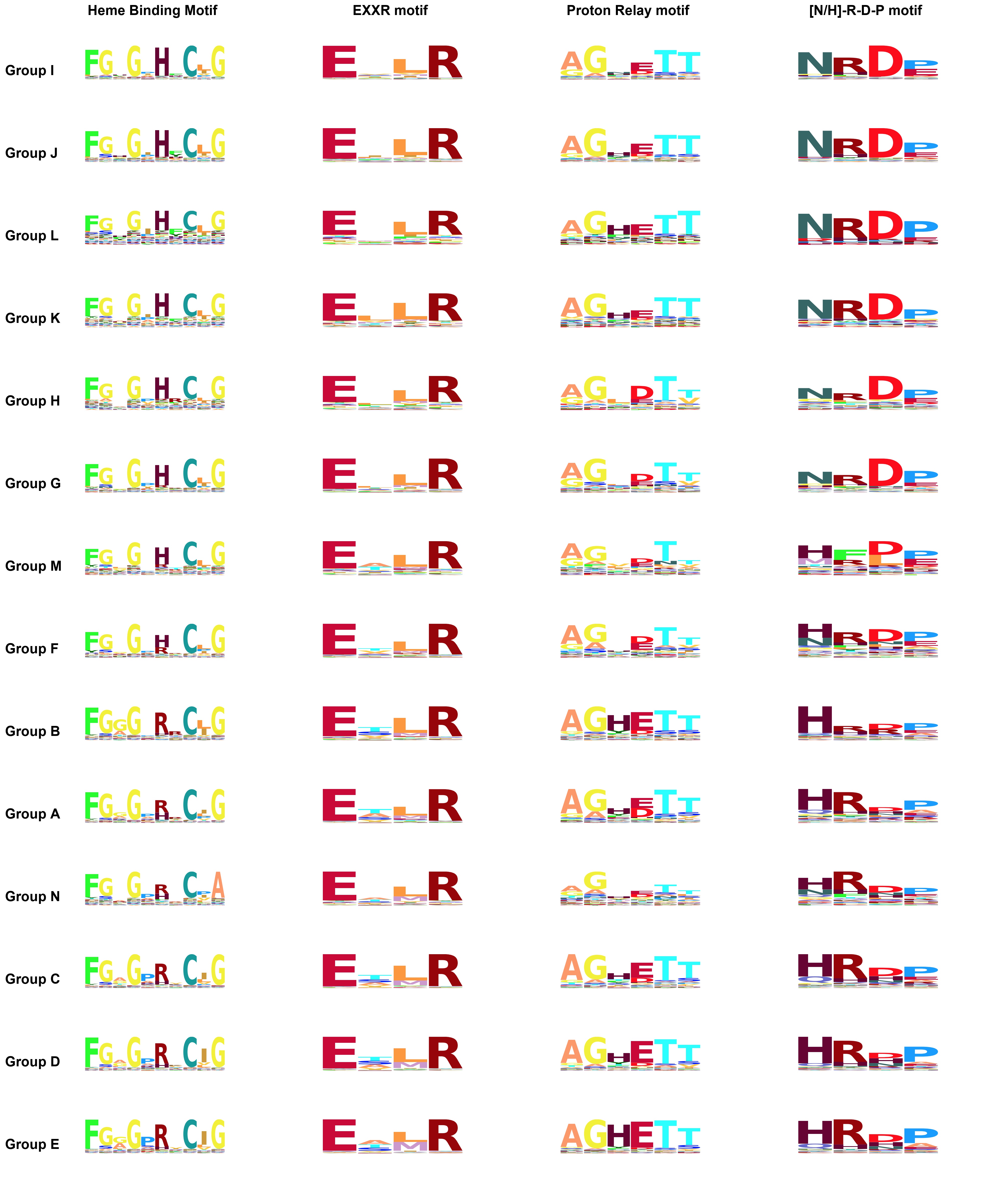

Supplement: Supplementary file 3 [file Image_1.JPEG]
